# The Quenching and Sonication Effect on the Mechanical Strength of Silver Nanowires Synthesized Using the Polyol Method

**DOI:** 10.3390/molecules26082167

**Published:** 2021-04-09

**Authors:** Junaidi Junaidi, Muhamad Wahyudi Saputra, Roniyus Marjunus, Simon Sembiring, Sutopo Hadi

**Affiliations:** 1 Department of Physics, Faculty of Mathematics and Natural Sciences, Universitas Lampung, Bandar Lampung 35145, Indonesia; mwsaputra31@gmail.com (M.W.S.); roniyus.1977@fmipa.unila.ac.id (R.M.); simon.sembiring@fmipa.unila.ac.id (S.S.); 2 Instrumentation Research Group, Department of Physics, Faculty of Mathematics and Natural Sciences, Universitas Lampung, Bandar Lampung 35145, Indonesia; 3 Department of Chemistry, Faculty of Mathematics and Natural Sciences, Universitas Lampung, Bandar Lampung 35145, Indonesia; sutopo.hadi@fmipa.unila.ac.id

**Keywords:** characterization, polyol, quenching, silver nanowires, synthesis

## Abstract

This study aims to determine the effect of fast cooling (quenching) on thermal properties, mechanical strength, morphology and size of the AgNWs. The synthesis of AgNWs was carried out at three different quenching-medium temperatures as follows: at 27 °C (ambient temperature), 0 °C (on ice), and −80 °C (in dry ice) using the polyol method at 130 °C. Furthermore, the AgNWs were sonified for 45 min to determine their mechanical strength. Scanning electron microscopy analysis showed that the quenched AgNWs had decreased significantly; at 27 °C, the AgNWs experienced a change in length from (40 ± 10) to (21 ± 6) µm, at 0 °C from (37 ± 8) to (24 ± 8) µm, and at −80 °C from (34 ± 9) to (29 ± 1) µm. The opposite occurred for their diameter with an increased quenching temperature: at 27 °C from (200 ± 10) to (210 ± 10) nm, at 0 °C from (224 ± 4) to (239 ± 8) nm, and at −80 °C from (253 ± 6) to (270 ± 10) nm. The lower the temperature of the quenching medium, the shorter the length and the higher the mechanical strength of AgNWs. The UV-Vis spectra of the AgNWs showed peak absorbances at 350 and 411 to 425 nm. Thermogravimetric analysis showed that AgNWs quenched at −80 °C have better thermal stability as their mass loss was only 2.88%, while at the quenching temperatures of 27 °C and 0 °C the mass loss was of 8.73% and 4.17%, respectively. The resulting AgNWs will then be applied to manufacture transparent conductive electrodes (TCEs) for optoelectronic applications.

## 1. Introduction

In the last decade, nanotechnology research has attracted the attention of researchers and academics in developing new technologies from materials that have good physical properties [1,2]. Nanotechnology is the manipulation of atoms and molecules to produce materials below the sub-macroscopic level [3,4]. The advantage of these nanostructured materials is the uniqueness of their mechanical, electronic, optical, and magnetic properties, which are different from those of micro or macro-sized materials [5,6]. However, an important factor in the development of nanotechnology is the synthesis of a material to obtain a nano-sized material.

The research on nanotechnology that is currently being developed is nanowires (NWs). NWs are one-dimensional nanostructures that have diameters of less than 100 nm and range in length from hundreds of nanometers to several micrometers [7]. NWs are considered to be promising materials because of their wide applications, such as in energy [7,8,9,10,11], environmental [12], health [13], sensor [14,15,16,17] and optoelectronics [17,18,19].

The synthesis of NWs requires the assistance of metal precursors. The metals that have were used are gold [20], silver (Ag) [21,22,23,24,25,26,27], and copper [28]. However, Ag is more widely used because of its high electrical and thermal conductivities, which are 6.3 10^7^ S m^−1^ and 429 W m^−1^ K^−1^, respectively [29,30,31,32,33]. Ag-based NWs (AgNWs) are well-established as transparent conductive electrodes [34,35,36].

Studies on the synthesis of AgNWs using the polyol method have been carried out by varying several other parameters, such as the oil bath temperature and the reaction time, the molecular weight of the capping agent, the molar ratio, the injection rate, the stirring rate, and the salt precursor; each parameter influences the size and properties of the AgNWs. One variation that has not been tried is that of the quenching medium during the final step of the synthesis process. Therefore, investigated the effects of three different quenching-medium temperatures, namely at 27 °C (in water), 0 °C (in ice), and −80 °C (in dry ice), on AgNW synthesis. The AgNWs were characterized using UV-Vis spectroscopy, scanning electron microscopy (SEM), transmission electron microscopy (TEM), differential thermal analysis (DTA), and thermogravimetric analysis (TGA).

## 2. Results

### 2.1. UV-Vis Analysis

**Figure 1:**
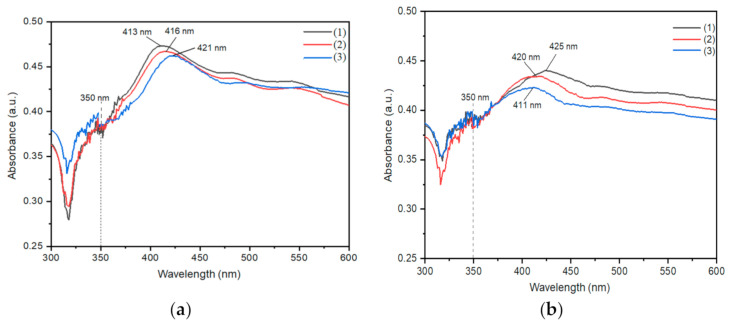
UV-Vis spectrum of AgNWs samples: (**a**) before being vibrated and (**b**) after being vibrated by (1) water medium, (2) ice medium, and (3) dry ice medium.

To analyze the optical properties of the AgNWs, UV-Vis spectroscopy has been utilized. Pure AgNWs are known to have a peak in the wavelength region between 350–390 nm [21,31,33,37,38,39]. The UV-Vis spectra of the AgNWs samples are shown in Figure 1.

### 2.2. SEM and TEM Analysis

**Figure 2:**
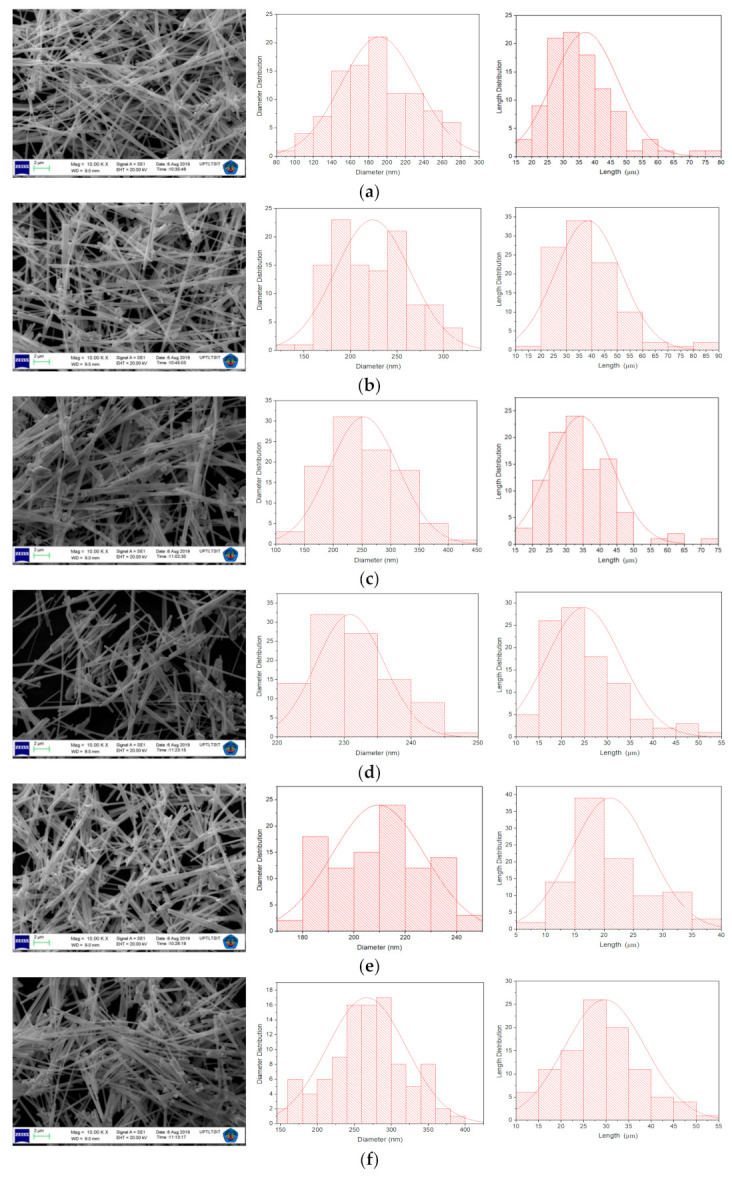
SEM images the morphology of AgNWs at quenching medium temperature (**a**) 27 °C, (**b**) 0 °C, (**c**) −80 °C, (**d**) 27 °C was vibrated, (**e**) 0 °C was vibrated, and (**f**) −80 °C was vibrated.

**Figure 3:**
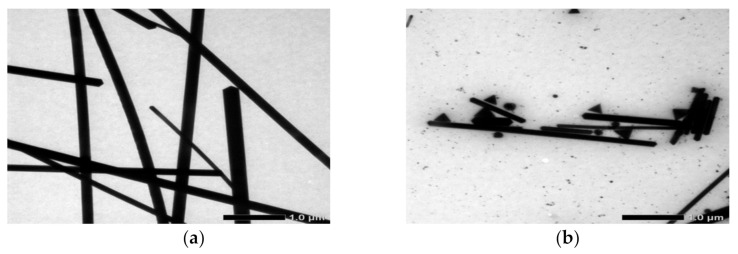
TEM images of AgNWs at quenching medium temperature 27 °C. (**a**) non-vibrated and (**b**) vibrated.

**Figure 4:**
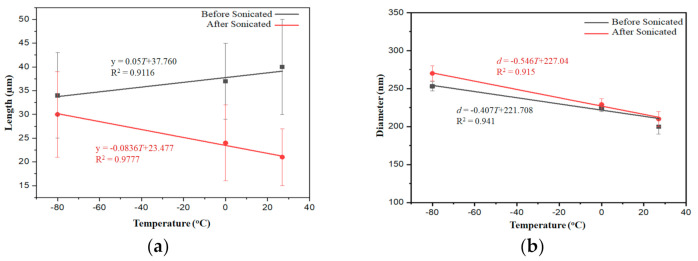
The relationship between the temperature of the quenching medium with (**a**) the length of AgNWs, and (**b**) the diameter of AgNWs.

SEM analysis was used to determine the morphology and size of the AgNWs. The samples that were characterized were samples from each variation of the temperature of the quenching medium and the vibrating effect using an ultrasonic cleaner. The SEM results were analyzed using the ImageJ application to find out the length and diameter of the AgNWs in each sample. The results of SEM characterization are shown in Figure 2. The TEM images and relationship between the temperature of the quenching medium of AgNWs are shown in Figure 3 and Figure 4.

### 2.3. DTA/TGA Analysis

**Figure 5:**
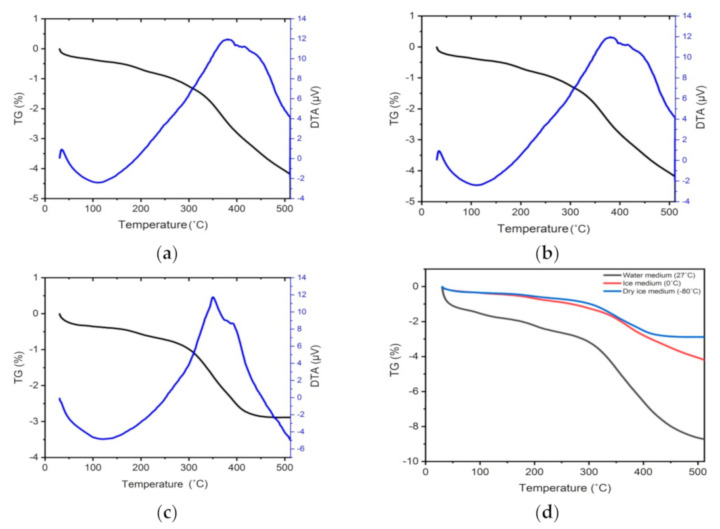
DTA/TGA AgNWs test results were quenched at temperature (**a**) 27 °C, (**b**) 0 °C, (**c**) −80 °C, and (**d**) comparison of the percentage of mass loss based on TGA characterization.

A thermal analysis of the AgNWs was performed using DTA/TGA. DTA/TGA analysis was carried out at a heating speed of 10 °C/min over a temperature range of 30 °C to 500 °C. The DTA/TGA analysis results are shown in Figure 5 and Table 1.

## 3. Discussion

From the UV-Vis analysis in Figure 1b, it is shown that all AgNW samples have two absorption peaks, namely a weak absorption peak at 350 nm and a strong absorption peak at 411 to 425 nm. These results indicate that the samples are not pure but rather consist of a mixture of AgNWs and AgNPs [28,29,30,31,32]. As observable in the spectra in Figure 1a, there is a shift in the wavelength (red shift) from 413 to 421 nm due to the increasing diameter of the AgNWs [40,41,42]. The decrease in absorbance shown in Figure 1 is caused by the decreasing length of the AgNWs due to the vibrational effect [37,43]. An additional broad shoulder appears in the peak above of 400 nm, which results from the distribution of possible orientations of the nanowires with respect to the incident light. Additional minor lifting of the red range of the spectrum is attributed to coupling to travelling plasmons along the nanowire and coagulation of particles, also known from spherical nanoparticles [16].

As seen in Figure 2, it appears that all AgNW samples produce the shape of NWs and some surrounding nanoparticles, and produce different lengths and diameters. These results are in accordance with the UV-Vis spectra shown in Figure 1. AgNWs synthesized at different quenching-medium temperatures are shown in Figure 2a–c. AgNWs quenched at 27 °C had a length and diameter of (40 ± 10) µm and (200 ± 20) nm, at 0 °C of (37 ± 8) µm and (224 ± 4) nm, and at −80 °C of (34 ± 9) µm and (253 ± 6) nm, respectively. These results indicate that a decrease in the average length of the AgNWs is caused by a decrease in the temperature of the quenching medium.

Figure 2d–f shows the results for AgNWs synthesized at different quenching-medium temperatures after the vibration test. A vibration test was carried out to determine the mechanical strength of the AgNWs. The results show that AgNWs synthesized at 27 °C had a length and diameter of (21 ± 6) µm and (210 ± 10) nm, at 0 °C of (24 ± 8) µm and (239 ± 8) nm, and at −80 °C of (30 ± 9) µm and (270 ± 10) nm, respectively. Based on these results, one can assume that the vibration test caused breakages in the AgNWs, which in turn rendered them shorter in length. However, the change in AgNW length in each sample was different; the largest change in length occurred in AgNWs quenched at 27 °C, while the smallest occurred in AgNWs quenched at −80 °C.

Figure 3 shows the TEM results of AgNWs synthesized after quenching at room temperature (27 °C). In the figure, it can be seen that the AgNWs before and after being vibrated experience a change in size in both diameter and length. AgNWs after being shaken, the length will decrease even if the many are destroyed. AgNWs quenched at 27 °C had a length and diameter of 30–50 µm and 180–220 nm (Figure 3a). After vibrating, the length and diameter of AgNWs became 5–10 µm and 190–230 nm (Figure 3b). The length of AgNWs after vibrating was below 1 µm. This condition can be said that the vibrating effect can destroy AgNWs and some even become AgNPs and AgNRs. The molecular weight and molar ratio of (PVP:Ag) are very important for controlling the growth and properties of the silver nanowires. The higher molecular weight of PVP, the greater diameter and length of silver nanowires [44].

Figure 4 shows the relationship between the temperature of the quenching medium and the length and diameter of the AgNWs. Figure 4a shows that the lower the temperature of the quenching medium, the lower the length of the AgNWs. The relationship between the temperature of the quenching medium and the length of the AgNWs can be described by the line equation. Furthermore, the lower the temperature of the quenching medium, the higher the mechanical strength of the AgNWs, as seen in Figure 4a. Based on Figure 4a, it appears that after the sonication there was a substantial change in AgNW length with increasing temperature of the quenching medium. Figure 4b shows that the lower the temperature of the quenching medium the higher the AgNW diameter. The relationship between the temperature of the quenching medium and the diameter of the AgNWs can be described by the line equation in Figure 4. The sonification caused an increase in diameter, but the change is not significant [45].

Figure 5 shows the results of the thermal analysis of the AgNWs (DTA/TGA curve). The DTA results for all AgNW samples did not differ, that is all samples showed an endothermic peak at a temperature of around 110 °C, which indicates the ethanol evaporation as a dispersant, and an exothermic peak at a temperature of around 360 °C, which indicates the melting and decomposition of PVP. The endothermic and exothermic peaks are followed by the mass loss observed in the TGA curve and can be seen in Table 1. These results indicate that during the endothermic process, namely the ethanol evaporation process, the AgNWs quenched at 27 °C experienced a mass loss of 3.94%, while at the quenching temperatures of 0 °C and −80 °C, the mass loss was of 1.54% and 1.35%, respectively. This was also followed by mass loss during the exothermic process, that is the polyvinyl pyrolidone (PVP) melting process. In this case, a lower mass loss was observed when the temperature of the quenching medium was lower; AgNWs that were quenched at 27 °C underwent a mass loss of 4.71%, while at 0 °C and −80 °C the mass loss was of 2.53% and 1.53%, respectively.

Figure 5d shows the comparison between the mass loss percentages in all three medium samples, i.e., water, ice, and dry ice. The results indicate that the larger total mass loss occurred in the AgNWs sample quenched at 27 °C (8.73%), while AgNWs that were quenched at 0 °C experienced a total mass loss of 4.19% and those quenched at −80 °C experienced a total mass loss of 2.88%. In Figure 5d it can be seen that the lower the temperature of the quenching medium the smaller the percentage of total mass loss of the AgNWs, which means that the thermal stability increases. This is in agreement with the SEM results showing that the lower the temperature of the quenching medium, the higher the mechanical strength of the AgNWs [46,47,48].

## 4. Materials and Methods

### 4.1. Materials

The materials used in this study were silver nitrate (AgNO_3_) 99% (Sigma-Aldrich Pte. Ltd., Science Park Drive, Singapore), PVP Mw. 55,000 g/mol (Sigma-Aldrich Pte. Ltd., Science Park Drive, Singapore), ethylene glycol (EG) 99.5% (Merck kGaA, Darmstadt, Germany), propylene glycol (Merck kGaA, Darmstadt, Germany), ethanol 96% (Merck kGaA, Darmstadt, Germany), FeCl_3_.6H_2_O (Sigma-Aldrich Pte. Ltd., Science Park Drive, Singapore) 0.04 M, silicon oil, deionized water, ice, and dry ice.

### 4.2. Synthesis

**Figure 6:**
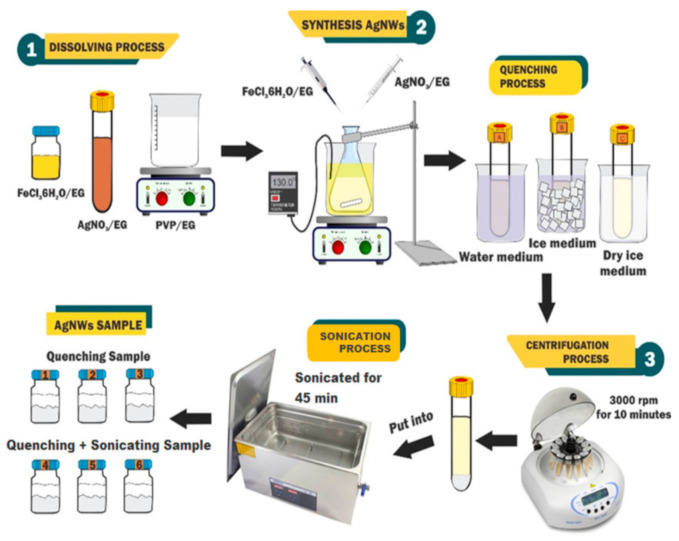
Synthesis of silver nanowires by polyol method.

The synthesis of AgNWs by the polyol method occurs in three stages, namely the dissolution process, AgNWs synthesis, and centrifugation [6,48], as shown in Figure 6. The AgNWs synthesis was initiated by dissolving 2.994 g of PVP into 60 mL EG in an Erlenmeyer flask at 130 °C for 1 h. Then, 4 µL of 0.04 M FeCl_3_.6H_2_O was added followed by the dropwise addition of 24 mL of 0.3 M AgNO_3_/EG for 2 h using a drop syringe with stirring at 350 rpm for 2 h. Then, the AgNWs samples were quenched in three different media, namely water, ice, and dry ice, and prickly heat to room temperature. After, the samples were washed with ethanol through a centrifugation tool for 10 min, and then a vibration test was performed for 45 min using an ultrasonic cleaner [27].

### 4.3. Characterization

AgNWs physical parameter tests include tests of optical properties, morphology and size, and thermal properties. The optical properties test was carried out using a UV-Vis spectrophotometer (Shimadzu, UV-1700, Japan) at a wavelength of 300–800 nm. The morphological and size tests of the AgNWs were carried out using Scanning Electron Microscopy (SEM, JEOL JSM-6510, Japan) at a voltage of 20 kV. To further ascertain the size of the AgNWs, Transmission Electron Microscopy (TEM, JEOL 2010, JEOL, Tokyo, Japan) characterization was carried out at a voltage of 120 kV. Furthermore, the thermal properties test to determine the mechanical strength of AgNWs was carried out using Differential Thermal Analysis (DTA) and Thermogravimetric Analysis (TGA) of type DTG-60 (Shimadzu, Kyoto, Japan) from 0 to 500 °C.

UV-Vis spectroscopy was used to determine the peak absorption of the AgNWs with a wavelength of 300–600 nm. SEM was used to determine the morphology and size of the AgNWs; the data was then analyzed using ImageJ software (open source developed by NIH and University of Wisconsin). DTA/TGA was performed to determine the thermal stability of the material. The temperature used was 500 °C to room temperature.

## 5. Conclusions

The effect of sonification or vibration on AgNWs from UV-vis results has a peak shift toward the red shift. The DTA results show that AgNWs have endothermic peaks at 110 °C and exothermic peaks at 350 to 380 °C, and that the best thermal stability occurs in AgNWs quenched at −80 °C. The quenching process in AgNW synthesis affects the length and diameter of the AgNWs. The lower the temperature of the quenching medium, the higher the mechanical strength and diameter of the AgNWs. The lower the temperature of the quenching medium, the smaller the percentage of total mass loss of the AgNWs. Here, we observed that the lower the temperature of the quenching medium, the shorter the length and the longer the diameter of the AgNWs.

## Figures and Tables

**Table 1:** DTA/TGA characterization.

Medium	Temperature (°C)	Heat Process	Loss Mass (%)
Water	30–330	Endothermic	3.94
	330–500	Exothermic	4.71
Ice	30–330	Endothermic	1.54
	330–500	Exothermic	2.53
Dry Ice	30–330	Endothermic	1.35
	330–500	Exothermic	1.53

## Data Availability

The data presented in this study are available on request from the corresponding author.

## Sample Availability

Not available.
